# EEG-Neurofeedback as a Tool to Modulate Cognition and Behavior: A Review Tutorial

**DOI:** 10.3389/fnhum.2017.00051

**Published:** 2017-02-22

**Authors:** Stefanie Enriquez-Geppert, René J. Huster, Christoph S. Herrmann

**Affiliations:** ^1^Department of Clinical and Developmental Neuropsychology, Faculty of Behavioural and Social Sciences, University of GroningenGroningen, Netherlands; ^2^Department of Psychology, Faculty of Social Sciences, University of OsloOslo, Norway; ^3^Experimental Psychology Laboratory, Department of Psychology, Faculty VI Medical and Health Sciences, University of OldenburgOldenburg, Germany

**Keywords:** neurofeedback, protocol tutorial, EEG, frequency band modulation, fm-theta, cognitive enhancement

## Abstract

Neurofeedback is attracting renewed interest as a method to self-regulate one’s own brain activity to directly alter the underlying neural mechanisms of cognition and behavior. It not only promises new avenues as a method for cognitive enhancement in healthy subjects, but also as a therapeutic tool. In the current article, we present a review tutorial discussing key aspects relevant to the development of electroencephalography (EEG) neurofeedback studies. In addition, the putative mechanisms underlying neurofeedback learning are considered. We highlight both aspects relevant for the practical application of neurofeedback as well as rather theoretical considerations related to the development of new generation protocols. Important characteristics regarding the set-up of a neurofeedback protocol are outlined in a step-by-step way. All these practical and theoretical considerations are illustrated based on a protocol and results of a frontal-midline theta up-regulation training for the improvement of executive functions. Not least, assessment criteria for the validation of neurofeedback studies as well as general guidelines for the evaluation of training efficacy are discussed.

## Introduction

**Figure 1:**
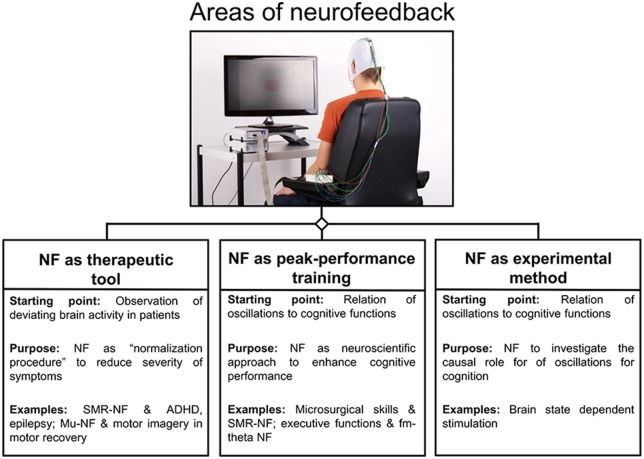
**Areas of neurofeedback application.** An overview of three main areas is given for neurofeedback applications, namely neurofeedback as therapeutic tool, peak-performance training and experimental method. For each area, the rationale behind is given and protocols are listed as examples (Abbreviations: NF = neurofeedback; Arns et al., 2009; Ros et al., 2009).

Based on recent methodological and technical progress, as well as on an increasing knowledge about the neural correlates of behavior and cognition, brain-computer interfaces (BCIs) for neurofeedback are attracting growing interest in both the scientific and medical communities as a method to self-regulate one’s own brain activity. Currently, neurofeedback can be used in at least three main ways: (i) as a therapeutic tool to normalize patients’ deviating brain activity in order to influence symptoms (e.g., motor learning in post-stroke recovery (Pfurtscheller and Neuper, 2006) or in attention deficit hyperactivity disorder (ADHD) or epilepsy (Monastra et al., 2002; Egner and Sterman, 2006; Birbaumer et al., 2009; Arns et al., 2013); (ii) as so-called peak-performance training to enhance cognitive performance in healthy participants (see review of Gruzelier, 2014a); and (iii) as an experimental method to investigate the causal role of specific neural events (such as brain oscillations) for cognition and behavior (see Figure 1) which is known as brain-state dependent stimulation (BSDS; e.g., Jensen et al., 2011; van Schie et al., 2014; Guhathakurta and Dutta, 2016; Royter and Gharabaghi, 2016).

**Figure 2:**
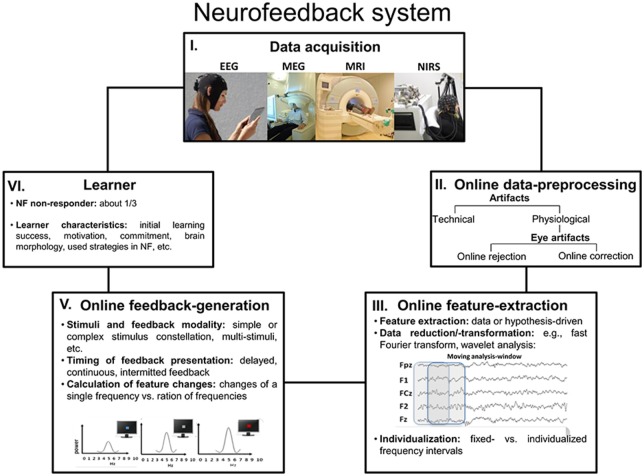
**Neurofeedback system.** This graphic outlines a summary over the five most important processing steps and parts that altogether constitute a neurofeedback system (Abbreviations: NF = neurofeedback).

BCIs rest on the measurement of brain activity and produce signals that are often directed at assisting, enhancing or repairing cognitive or sensory-motor functions. Here, open-loop applications can be dissociated from closed-loop designs. In the former, brain activity is recognized by the computer and used as a command, for instance to assist the participant to interact with the environment (Birbaumer et al., 2009; Millán et al., 2010), or it is used as a trigger for stimulus presentation in BSDS (e.g., Jensen et al., 2011). In closed-loop applications, a sensory representation of brain activity is fed back to the user in real-time with the goal of assisting self-regulation of brain activity; this form is known as neurofeedback. In case of closed-loop BSDS, the monitoring of brain activity can be used to guide the application of brain stimulation. Hereby the actual stimulation depends on specific brain states (Hartmann et al., 2011; Schestatsky et al., 2013). Closed-loop neurofeedback applications are implemented by a software system and a processing pipeline, altogether consisting of five elements (see Figure 2).

### The Five Elements of a Neurofeedback Processing Pipeline

In short, the **data acquisition of brain signals (1)** can be performed using different methods, such as high temporal resolution electroencephalography (EEG) and magnetoencephalography (MEG), which are optimal for real-time feedback of brain processes. Besides those, high spatial-resolution functional magnetic resonance imaging (fMRI) and near-infrared spectroscopy (NIRS) are also increasingly used (Please note that EEG neurofeedback for brain oscillations represents the topic of the current report). The next element of a neurofeedback system is the **online data-preprocessing (2)** and the major task at this step is the detection and rejection or correction of artifacts, of which eye and muscle artifacts are most common. Various artifacts generate activity that affects the whole EEG frequency spectrum, including those frequencies that are usually the focus of neurofeedback training. In the worst case, for example when ignoring eye artifacts, the participants may falsely learn to modulate their eye movements rather than their brain activity. The so-called **feature-extraction (3)** stage concerns the selection and extraction of features computed from brain activity that are used during neurofeedback; usually, these features represent that pattern of brain activity that one wants to modulate. This may simply translate to the selection of a specific frequency band of the EEG, which corresponds to the “working language” of a brain network associated with a specific cognitive function. However, more sophisticated procedures relying on machine learning algorithms or advanced techniques for data decomposition are suitable as well, although these are currently not commonly used. Quite obviously and as stated by Zich et al. (2015), suboptimal feature extraction will cause reduced neurofeedback success, because a sub-optimally designed feature extraction will not capture the brain activity of interest. The **generation of a feedback signal (4)** converts the characteristics of the extracted feature into a sensory stimulus that can be presented to and be processed by the learner. The feedback signal thus indicates the activity state of the targeted brain system or process in relation to a criterion, and signals when the targeted characteristic or feature of brain activity meets a specific threshold or state. In contrast to other methods for neuromodulation, such as electrical simulation, with neurofeedback the **learner (5)** is actively engaged, constantly applying and adapting strategies to alter his/her brain activity in the intended direction. Learner characteristics that determine the success of neurofeedback training have become the focus of attention recently. In a special issue by Friedrich et al. (2014), learner specific aspects such as positive mood states (Subramaniam and Vinogradov, 2013), motivation (Kleih and Kübler, 2013), locus of control (Witte et al., 2013), all turned out as being relevant for the prediction of individual learning success in specific neurofeedback protocols. However, evidence also suggests that the gross-morphology of brain areas generating EEG features used for neurofeedback training may be associated with training success (Enriquez-Geppert et al., 2013; Halder et al., 2013; Ninaus et al., 2015). This topic is of direct consequence for personalized interventions, where learner characteristics may be used to assign participants to interventions to which they are most likely to respond. Weber et al. (2011), for example, developed a classification scheme to determine neurofeedback responders and non-responders at an early stage of training. In case of BSDS, the participant is not regarded as a learner, instead a **stimulator device (5)** is adapted online to either present an experimental stimulus (e.g., Kruglikov and Schiff, 2003) or apply external stimulation (e.g., electrical or magnetic see Otal et al., 2016).

In the following, we will discuss three aspects concerning the conceptualization of neurofeedback learning: operant conditioning in the context of neurofeedback, neurofeedback learning in the context of control-theoretical models, and neurofeedback in the context of the dual-process theory.

### Neurofeedback Learning

Operant (or instrumental) conditioning is considered as the principle learning mechanism underlying the self-regulation of brain activity via neurofeedback. Generally, operant conditioning states that the probability of a future response is dependent on its association with an immediately following consequence; positive consequences increase the likelihood of given behavior, whereas negative consequences decrease it. Changing the brain’s activity through such conditioning is not genuinely new. As early as Fetz (1969) made use of operant/instrumental conditioning to enhance cortical single cell activity of the pre-central motor cortex, showing that macaca mulatta monkeys could learn to self-regulate their neural activity. Similarly, instrumental conditioning of intracranial EEG over the sensorimotor cortex, measuring synchronized local field potentials (LFP), led to self-regulation of different EEG patterns in the cat (Sterman and Wyrwicka, 1967). On a macroscopic scale and based on scalp EEG, Kamiya was one of the first to show that humans were able to self-regulate their activity in the alpha frequency band (8–12 Hz) by instrumental/operant conditioning (Kamiya, 1968), and Sterman et al. (1974) were among the first to apply neurofeedback as a therapeutic tool in epilepsy (Sterman and Friar, 1972).

Neurofeedback learning has recently also been outlined based on a control-theoretical framework. This framework considers the above mentioned closed-loop pipeline and details a sequence of learning events form a neural perspective. Ros et al. (2014) describe the initial neurofeedback stage as mainly characterized by fluctuating feedback signals that reflect stochastic and unconditioned neural variability. On following fortuitous events, it is supposed that neural variability will then infrequently generate activity that meets the threshold for reward. As soon as the sensory representation of this above-threshold brain-activity is followed by a rewarding feedback signal, the brain is able to memorize this distinct neural/behavioral state as a so-called internal set-point. This elicits a reward-modulated signal (such as dopamine) that supports synaptic plasticity. Subsequent feedback-loops aim at the reproduction of this set-point by using strategies in a feedforward way, thus comparing the actual state with the target state. Multiple loop-iterations (conditioning trials) will then lead to further refinement of the set-point, and to a more efficient strategy for its reproduction.

In addition, cognitive factors have also been shown to influence neurofeedback learning. Wood et al. (2014) provided a framework based on the dual/two-process theory, which initially was introduced in the more general context of biofeedback (see also, LaCroix, 1986). This theory dissociates automatic from controlled processes, which have different characteristics. Whereas automated processes are regarded capacity-free, unconscious and difficult to control by self-instruction, controlled processes reflect capacity-limited activity of the supervisory attention system (Shallice and Cooper, 2011) and are mainly regulated by self-instruction. Furthermore, while automated processes are acquired based on reward-learning, controlled processes are mainly driven by direct self-instruction. On the basis of this theory, Wood et al. (2014) suggest the existence of three networks that rely on either a single or a mixture of both types of mental activity. Central for neurofeedback learning, propose the so-called “local control network” that encompasses specific automatic processes (which are influenced by the feedback signal) and controlled processes (e.g., verbalizations and self-instructions), which are both necessary for the specific neurofeedback context. Automatic processes irrelevant or hindering in context of neurofeedback (such as rumination) are subsumed under the “organismic control network”. Controlled processes not aiding neurofeedback learning (such as improper strategies) are ascribed to the “central control network”. An optimal state for neurofeedback learning is reached when irrelevant associations between internal states and external reward are avoided, and when the learner stays engaged, focused and undistracted. Wood et al. (2014) therefore suggest the monitoring of inner speech via associated brain activity during neurofeedback to provide additional feedback signals when the learner should reduce excessive attention towards himself.

### Neural Communication Mechanism as the Target of Neurofeedback

To infer a robust and reliable control signal, neurofeedback should be approached as a hypothesis-driven application based on interdisciplinary knowledge from neuroscience, psychology and neuropsychiatry (Jensen et al., 2011; Horschig et al., 2014). The understanding of the physiological basis of neural oscillations led to recognizable advancements in recent years (Wang, 2010). Neural oscillations have been observed throughout different levels of neural organization, ranging from single-neuron activity as subthreshold membrane potential oscillations and action potentials, to local activity of assemblies of neurons, and even to activity patterns of whole cortical networks in context of different brain areas (e.g., Akam and Kullmann, 2012; Buzsáki et al., 2013). Oscillatory activity of neural populations has been suggested to represent a major communication mechanism of the brain (Buzsáki et al., 2013) and has furthermore been related to cognitive functions (Basar et al., 1999; Herrmann and Knight, 2001). Consequently, abnormal oscillatory activity has been associated with psychiatric and psychological disorders such as ADHD, Alzheimer’s disease, schizophrenia, bipolar disorder, or mild cognitive impairment (e.g., Başar, 2013; Başar and Güntekin, 2008; Başar et al., 2016). Electrophysiological studies of the normal functioning of basal ganglia-thalamocortical circuits and the pathophysiology of Parkinson’s disease provided insights into the functional role of neural oscillations (Schnitzler and Gross, 2005). In the following, the associations between oscillations and cognition will be elucidated using the example of theta oscillations and executive functions. We introduce this association in more detail here, since theta oscillations will later be used as reference for the discussion of the various choices and options available for the design of a neurofeedback protocol.

Theta oscillations have been shown to emerge as predominant activity from different brain areas including the hippocampus and the midcingulate cingulate cortex (MCC; e.g., Womelsdorf et al., 2010a). Theta oscillations have furthermore been suggested to reflect a common mode for communication of local computations within larger networks (Buzsáki, 2006; Wang, 2010). Their physiological characteristics may indeed enable the grouping and segregation of neural assemblies and the assignment of various computational tasks to them (Buzsáki, 2002). For instance, extracellular measures in animal models demonstrated that neural representations of relevant stimulus-response mappings are organized in time by theta activity of single neurons by the interplay of the MCC and the prefrontal cortex (PFC; e.g., Johnston et al., 2007; Womelsdorf et al., 2010b). Here, a pro-/antisaccade task switching either required a switch between two task rules, or implied the continuation of the previous stimulus-response assignment. LFP oscillations in the MCC only translated to selective firing rates of neuronal groups that represented one or the other task rule when there was actually an immediate switch. In contrast, selective PFC neurons signaled the task rule only after some trials once there was a switch, and thus when the new task rule had been established (Johnston et al., 2007). In a similar experiment, Womelsdorf et al. (2010b) demonstrated that theta activity in the MCC could in fact predict the specific response the monkey was about to produce. Task rules were selectively coded in theta activity of spatially separate neuronal groups and task-selective theta activity emerged early in trials requiring adjustments of task rules. Womelsdorf et al. (2010b) thus suggested that the degree with which individual nodes, e.g., the MCC, functionally contribute to the theta network depends on given task demands. Their contribution is high, for instance, when executive control is needed for the re-establishment of a task rule. Without further task demands, rules may be kept up by theta-based somato-dendritic activation, but this local excitation would not be sufficient to spike output to the externally generated rhythmic modulations of excitability. Recently, Colier et al. (2016) reported an up-regulation theta neurofeedback study based on intracranial recordings in humans.

**Figure 3:**
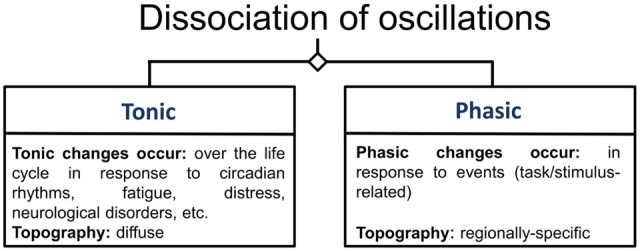
**Categorization of oscillations.** This figure illustrates the categories of oscillations according to Klimesch (1999). Oscillations are differentiated according to whether they are measured during rest, in which case they are labeled tonic oscillations, or whether they are related to specific task-conditions or stimuli, under which condition they are referred to as phasic oscillations.

Frontal-midline theta (fm-theta) does not reflect band-pass filtered event-related potentials (ERPs) or other non-oscillatory transients (Cohen and Donner, 2013) and is constituent of phasic responses to events that require attention and cognitive processing. In contrast to tonic oscillations, which show a diffuse topography, such phasic oscillations reflect changes in oscillatory activity in response to an event and exhibit a specific topography (Klimesch, 1999; see Figure 3), in this case a maximum amplitude at frontal and central scalp electrodes (Ishihara et al., 1981). The MCC seems to be a dominant source of this EEG phenomenon (Gevins et al., 1997; Asada et al., 1999; Sauseng et al., 2007). Fm-theta power is increased when cognitive processing is enhanced (Mitchell et al., 2008), and the absence of fm-theta up-regulation in response to demanding tasks seems to be associated with reduced task performance in healthy (Donkers et al., 2011) as well as patient groups (Schmiedt et al., 2005). In a study applying exogenous theta oscillations in form of transcranial alternating current stimulation (tACS) to a mid-frontal scalp region during the performance of an executive functioning task, tACS led to improved behavioral performance when compared to alpha band tACS. This result thus supports the idea of fm-theta as causally contributing to executive functioning (van Driel et al., 2015).

However, neurofeedback can also be used to gain further knowledge about basic neurocognitive functioning, for instance by investigating the relevance of oscillatory features, such as the amplitude or phase, for cognition and behavior. For instance, van Schie et al. (2014) investigated the effects of controlled fm-theta down- and up regulation on working memory performance, and Gho and Varela (1988) assessed the relevance of the phase of alpha oscillations for the perception of visual stimuli.

Many neurofeedback protocols exist that target different neuronal phenomena observed in EEG measurement. Such protocols differ regarding the frequency band addressed (e.g., alpha-, beta, theta-, gamma-training), the utilization of different electrode locations (Fz, Cz, Fz1, etc.), and the recording of the EEG under different activity states of the subjects, e.g., eyes-open or eyes closed (Gruzelier, 2014a). Based on findings about hippocampal theta and its relation to memory, for example, a theta-upregulation neurofeedback at electrode Pz was performed which indeed led to improved memory consolidation (Reiner et al., 2014). Notably, different protocols can influence varying brain networks as long as they rely on biologically relevant frequencies (Hutcheon and Yarom, 2000). A protocol can be considered operational, if the EEG is modulated in accordance with instructions, even though such changes might not always be accompanied by cognitive or behavioral changes; the latter, however, usually is the aim of most neurofeedback studies.

## A Neurofeedback-Protocol: Step-By-Step Guidelines

### Training Design

The foundation of each neurofeedback study is its design (e.g., a pre-post measurement design), which usually should include an experimental and an active control group (for more information see Grimshaw et al., 2000). The implementation of an active control group enables not only the control of repetition-related effects (here a passive control group would be sufficient), but also for non-specific effects that may be caused by the overall setting (e.g., Campbell and Stanley, 1963). The demonstration of outcomes in accordance with known associations of a specific oscillation (with e.g., a specific cognitive process or a symptom) is a convincing demonstration of the specificity of the training, especially when using neurofeedback as an experimental method to investigate the causal role of oscillations.

Regarding the realization of feasible active control groups, different possibilities exist. One of those is the instantiation of a pseudo-neurofeedback condition, in which a given participant of the control group receives a replay of the feedback signal derived from a matched participant of the actual experimental group (e.g., as used in Enriquez-Geppert et al., 2013). Importantly, such a feedback-replay can be combined with the online monitoring of artifacts from the control subjects, such that blinks may disrupt the feedback replay, thereby indicating to the control subject that indeed activity is recorded (although it may not be used for the actual training). The reactivity of the feedback-system to the participant’s behavior (here in terms of observable or physiological artifacts) strongly enhances the credibility of the control condition. Such a control condition will be most efficient with short-latency feedback, i.e., the feedback signal is quickly following the recording and extraction of the neural feature (e.g., in the order of few 100 ms). However, if the feedback signal is being computed on the aggregate performance over larger time spans (e.g., a block of 30 consecutive seconds for example), replaying the feedback signal of a matched subject of the experimental group to a control subject may not completely dissociate feedback from brain activity; at least initially control subjects will follow instructions and may achieve the requested modulations of brain activity over certain time periods. Thus, theoretically there may still be some contingency between brain activity and feedback signal. However, aggregating over longer time periods also increases the delay between brain state and feedback signal, which in itself should decrease the efficacy of a learning process.

Another feasible option for the realization of an active control group might be to train another frequency band than the actual feature of interest (e.g., Reiner et al., 2014). For instance, while the experimental group is intended to enhance their theta activity in order to improve executive functioning, the active control group might learn to enhance their beta activity for which there is little evidence that it relates to executive functions. A variation may be to base feedback on a different frequency band for every new training session (e.g., session 1: feedback based alpha; session 2: feedback based on gamma-; Session 3: feedback based on delta- activity, etc.), which should effectively prevent strong frequency specific learning.

A further possibility for the implementation of an active control group is inverse feedback. Here, different feedback blocks or groups may use different instructions with respect to the modulation of the very same feature; this was, for example, implemented by van Schie et al. (2014), who trained both theta up and down regulation, thereby observing also opposite behavioral changes.

Whatever the exact implementation of the control condition is, its credibility is an important issue in context of factors such as learned helplessness (Seligman, 1975; Abramson et al., 1978) and resentful demoralization (Onghena, 2005). Learned helplessness describes passive behavior as a consequence of the learners’ realization that nothing they do has any effect on the training outcome. Resentful demoralization describes negative behavioral effects (non-compliant or uncooperative behavior etc.) that may occur when learners perceive their intervention as inferior or realize they are not part of the experimental condition.

In certain clinical setting, for instance with psychiatric groups, a within-subject ABA design incorporating a control condition rather than a second group is inevitable. In ABA designs, feedback is first given on the desired brain state during the “A” phase, and then for the reversed condition during phase “B”, before again feedback is given in the intended direction in the last “A” phase (e.g., a sequence of up-, down-, and up-regulation of an EEG feature such as fm-theta).

For BSDS as an experimental tool to investigate the functional role of oscillations, active control groups play a rather minor role. Here, a one session within-group design is sufficient to investigate specific features of oscillations and their association with behavioral performance (e.g., comparison of behavioral effects when a stimulus is presented at high amplitudes vs. low amplitude or within phase vs. out-of-phase).

**Figure 4:**
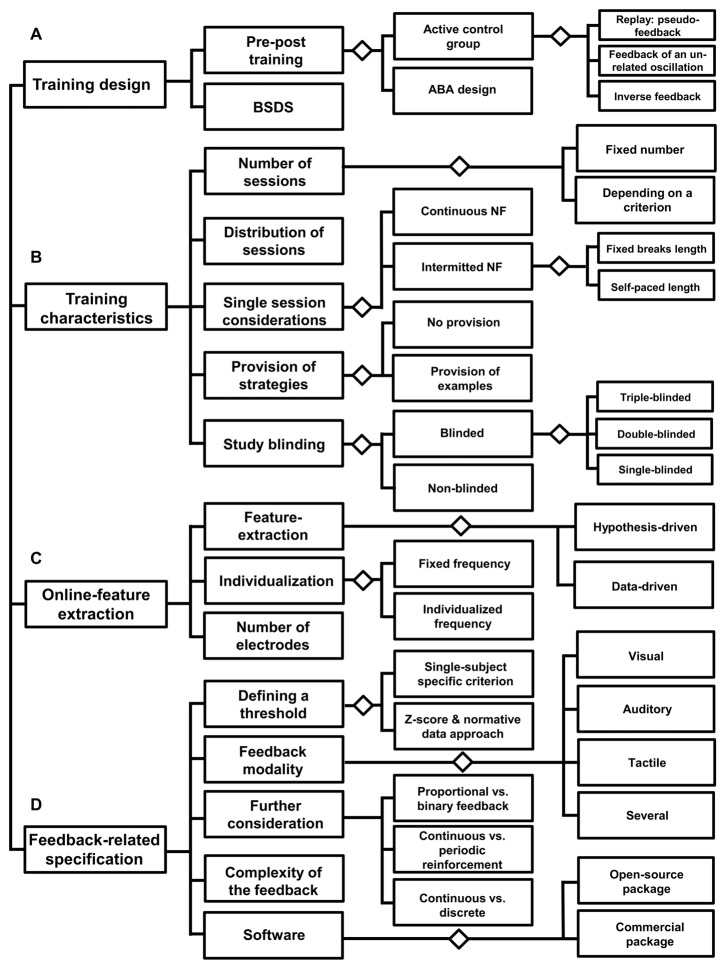
**Decision diagram: before neurofeedback implementation.** This diagram lists three areas **(A–D)**, which refer to the preparation of a neurofeedback intervention. Each of these areas contains aspects that should be considered in order to set-up an effective neurofeedback training design.

The decision diagram in Figure 4 should serve as a starting point to sketch a neurofeedback study. In the following, the paragraphs serve to go through all the important steps in detail.

### Training Characteristics

#### Number of Training Sessions

As the first step, the number of training sessions should be defined. It is most common to implement a fixed number of sessions based on effect sizes of similar protocols. However, a training goal could also be defined in terms of a specific performance pattern, for instance the reduction of a specific symptom as operationalized by values of a clinical questionnaire. In such cases, usually the number of sessions cannot be determined before the intervention. However, Strehl (2014) also discusses the possibility of overtraining, referring to the notion that a surplus of practice may actually decrease training efficacy. As a consequence, it might be necessary to monitor the learning curve of a given subject to individually adapt the number of training sessions.

#### Distribution of Neurofeedback Sessions Over the Whole Training

Another decision regards the distribution of single training sessions over the course of the whole training period; sessions have been conducted as often as twice a day, or only once a week. Research comparing massed and distributed learning has strongly focused in an educational school context (Carpenter et al., 2012). Here, one of the most reliable findings is an advantage for distributed learning in contrast to massed learning (Ebbinghaus, 1885/1913). In context of coordinated reset stimulation, which is a type of deep brain stimulation used in neurological and psychiatric disorders to unlearn abnormal neuronal synchrony, the spacing principle has been tested in a computational study again exhibiting an advantage of a spaced as compared to a massed intervention (Popovych et al., 2015). However, up to now there is little known about whether few or many neurofeedback sessions within a certain time interval are more helpful for learning to self-regulate brain activity, and less is known even regarding the length of an effective gap between training sessions.

#### Single Session Considerations

Further specifications concern the duration of a single session. Common durations for a single session are about 20–40 min, but this strongly depends on the participants’ ability to focus on the training, which differs across age groups and further varies with the participants’ health status. Not least, it should be decided whether neurofeedback should be performed continuously within a session or interrupted breaks. The duration and number of breaks can either be defined by the experimenter before study onset or by the participant during training.

#### Provision of Strategies

Regarding the relevance of strategies and instructions for neurofeedback outcome, little systematic research has been conducted thus far. It may be helpful to provide exemplary strategies for the participants on how to alter the brain activity of interest. A special case is neurofeedback for motor recovery in stroke patients. Overt and covert movements both elicit event-related desynchronization in mu (8–13 Hz) and beta frequency ranges over the scalp in sensorimotor cortical regions contralateral to the imagined part of the body (Pfurtscheller and Lopes da Silva, 1999; Cheyne, 2013). Thus, learning to regulate such desynchronization is thought to potentially translate into motor function recovery and participants are instructed to imagine specific motor movements. However, motor imagery has also been used in a new neurofeedback approach for the self-regulation of increased alpha band connectivity between the motor cortex and the rest of the brain (Mottaz et al., 2015). Regarding strategies in other domains of neurofeedback research, some researchers provide mental strategies for the subjects to start with (for instance participants in a theta-beta-feedback training are often instructed to relax and to be attentive), whereas others only roughly instruct participants to try whatever they find useful to self-regulate their brain activity. For a slow cortical potential study, Roberts et al. (1989) reported nothing such as a single valid strategy; instead they found high inter-individual differences in successful strategies. In a SMR study, participants could not even indicate a specific strategy.

However, regarding the provision of exemplary strategies, the phrasing of sufficiently detailed instructions at the beginning of a training session is a topic of current investigations also with respect to the learning outcome (Lotte et al., 2013). Davelaar et al. (2016), for instance, conducted a thematic analysis of verbal protocols to investigate differences between responders and non-responders in a single session of alpha upregulation training. The authors raised the question whether the relatively large proportion of non-responders may be caused by incongruently formulated instructions (regarding the desired outcomes), and stress the importance of well-articulated instructions.

#### Study Blinding

Thereafter, it should be specified, if the neurofeedback study should adopt a single- (the learner does not know if he belongs to the experimental or the control group), double- (both learner and researcher do not know about the exact group assignment) or even triple- (a third party assessing effects) blind study design. Specifically, neurofeedback studies have been criticized for not being adequately blinded (e.g., Micoulaud-Franchi et al., 2014), thereby neglecting non-specific factors, such as expectancy effects, and thus hindering the evaluation of the treatment effectiveness. The reasons for the lack of such blinded studies, however, should also be borne in mind. As Lansbergen et al. (2011) summarized specifically concerning neurofeedback as clinical intervention for children with ADHD, blinded studies are confronted with ethical issues of withholding treatment and are under pressure to develop highly feasible active control interventions. Issues regarding treatment efficacy and optimal set up of control condition are still not solved in context of ADHD as demonstrated by a recent meta-analysis (Cortese et al., 2016). Thibault and Raz (2016) argue that even most of clinical studies tend to be poorly designed and implemented. Based on these considerations, the importance of a reasonable control condition as discussed in the Section “Training Design” becomes even more evident.

### Online Feature-Extraction

#### Feature-Extraction

Next, the features of brain activity to be extracted in order to best test the research hypothesis need to be defined. In general, feature extraction can be performed in a data-driven manner, for instance with BCI aiming at the control of specific devices, such as a letter-spelling BCI (De Vos et al., 2014). However, with neurofeedback as a method to alleviate symptoms or enhance cognitive and behavioral performance, feature selection is usually based on evidence for an association between oscillations and cognition or symptoms. For instance, a feature could be specified by the identification of brain activity that differs between patients and healthy controls, aiming at an EEG profile that becomes more similar to healthy subjects. In addition, selected features can be measured at rest or during task processing. In the easiest, and most common case, EEG amplitudes of a given frequency band are extracted and averaged across one or several electrodes. Usually, these values are calculated relative to a baseline measurement. Similar to the design of a feedback signal, the underlying feature can also either directly reflect the activity of a single brain system or process, or be computed by putting the system of interest into context with another system. For example, activity from a single frequency band is often considered as a feature; however, the activity in a given frequency band can also be determined relative to changes within another frequency band (e.g., theta/beta ratio training). It also needs to be specified whether changes are calculated relative to a baseline measurement before the beginning of each single training session or whether a common baseline is used that is constant across all training sessions.

#### Individualization of Feature Extraction

Furthermore, the degree of individualization for feature extraction has to be determined. This, for example, refers to whether a fixed-frequency or an individualized-frequency interval should be used. An individually determined frequency-interval can, for example, be chosen by having subjects process experimental cognitive tasks and calculate the subject-specific dominant frequency peak by means of a frequency or time-frequency transform. With reference to the determination of the degree of individualization, it is assumed that neurofeedback will be more effective when relying on individualized features, since exact characteristics may vary across subjects as a function of age, disease states, task performance capabilities, or brain volume (Klimesch, 1999; Moretti et al., 2004). It should be noted that it is also possible to generate individual features in a more data-driven manner, e.g., through the application of machine learning algorithms. A spatial filter for fm-theta activity, for example, could also be generated through the application of independent component analysis or related procedures that provide means to decompose the recorded EEG into its generating latent sources. The training of a classifier and the application of its learned model for neurofeedback training should be applicable too, but would necessitate the identification of target states from a previous recording. Not least, features can also be adapted during training, e.g., through re-training of classifiers over the course of several neurofeedback sessions (e.g., Vidaurre et al., 2011; Bryan et al., 2013), or the adaptation of the threshold for positive feedback based on perceived task difficulty (Bauer et al., 2016). Thus, in principle many more sophisticated procedures for feature generation and extraction do exist, but few of them have actually been applied and systematically compared in the neurofeedback-based literature.

#### Number and the Location of Electrodes

Rogala et al. (2016) show that neurofeedback studies differ greatly regarding electrode positioning and the number of electrodes used for quantification of putatively the same brain feature. However, the feature extraction stage needs to be adapted to reflect interindividual differences in brain anatomy and function. It is known, for instance, that fm-theta may shift up to three centimetres around electrode Fz (Ishihara et al., 1981). Thus, the inclusion of several electrodes for the calculation of fm-theta is necessary to compensate for these shifts. Another reason to choose several adjacent electrodes would be to average across these in order to enhance the signal-to-noise-ratio. The selection of the number of electrodes depends certainly also on the type of neurofeedback application. Neurofeedback at the source level, for example, necessarily relies on a high number of electrodes to enable good localization estimates (see Song et al., 2015). With regard to a multi-session neurofeedback training as a therapeutic tool, for instance, a rather limited number of electrodes may be adequate for pragmatic reasons.

### Feedback-Related Specifications

#### Defining a Threshold

The feedback signal indicates the targeted brain activity process in relation to a criterion. Here, a variety of theoretically calculations are plausible for the definition of a specific threshold or state that can be based on changes relative to a resting condition, or relative to the mean or the median of a previous training session. These thresholds usually are calculated for single subjects. However, especially for clinical applications a different approach for the calculation of a threshold has been suggested. Here, thresholds for a given subject are not exclusively based on the subject’s own brain activity, but rather on the subject’s *z*-score relative to a normative sample. This reflects the idea to “normalize” the deviating EEG signature (Thatcher and Lubar, 2009). This procedure is known as *z*-score training and is related to a research line initiated since 1969 by Thalia Harmony and Roy John. This neurometrical approach provides an estimate of deviation by comparing single subjects to a large normative database of healthy subjects (Harmony, 1975, 1984; John et al., 1977, 1987; Hernandez-Gonzales et al., 2011).

In addition, feedback signals can be given in only or two directions, e.g., providing a reward signal when the brain activity exceeds the intended threshold, or by providing additional negative feedback when brain activity changes in the direction opposite to the intended one (e.g., Zoefel et al., 2011).

#### Feedback Modality

At this stage, a decision on the modality of the feedback signal has to be made (auditory, tactile, visual, combined modalities, etc.). Regarding motor imagery training, effects of different feedback modalities have been assessed. Ono et al. (2013) compared three types of visual feedback: (i) a simple bar changing its length; (ii) an animated hand changing its posture from open to a grasp (displayed at the subjects eye-level); and (iii) the same animated hand, but displayed at the subjects own hand position. All conditions led to enhanced event-related desynchronization over the contralateral sensorimotor cortex, but a stronger gain was observed with the third feedback type, where motor imagery and the feedback corresponded best. Moreover, Vukelic and Gharabaghi (2015) compared a visual feedback (movement of a cursor ball towards a target) with proprioceptive feedback using a brain-robot interface and investigated the effects on connectivity networks of coherent oscillations. They also observed an advantage for the proprioceptive condition, which led to increased volition control of brain activity compared to the visual condition. Regarding the self-regulation of slow cortical potentials as communication tool with completely paralyzed participants, superior effects were shown with visual feedback compared to auditory feedback (Hinterberger et al., 2004).

However, there are still too few systematic studies comparing the effects of different feedback modalities for specific protocols and specific populations. Decisions regarding the selection of the feedback modality are thus often based on practical considerations and learner specific characteristics. Basta et al. (2011), for instance, developed a vibro-tactile feedback as a vestibular rehabilitation program in daily life situations for elderly with the goal to reduce body sway in balance disorders, and reasoned that a tactile neurofeedback protocol could have higher efficacy than an auditory feedback, as potential sensory conflicts that feedback signals may be induced. More precisely, their protocol was designed to avoid the crossover of sensory input; tactile feedback, for example, avoids simultaneous vestibular stimulation effects as it would have resulted from auditory feedback (see Probst and Wist, 1990). The prevention of possible feedback-related effects on the vestibular system is of course of special important in the case of vestibular rehabilitation in balance deficits.

Fernández et al. (2016) had to take similar considerations into account when working with disabled children. This subject group is known to have a lower processing speed and shows difficulties in semantic processing in the visual but not in the auditory modality.

Some studies further utilized multimodal feedback signals. Kober et al. (2015), for example, evaluated the benefit of two neurofeedback protocols (SMR and upper alpha) as cognitive rehabilitation tools after stroke. They utilized a combined audio-visual feedback, in which a bar changed color from red to green in real-time when brain activity changed in the intended direction. In addition, they created a distinctive reward: participants received points as reward (a reward counter kept track and was continuously displayed), and an additional midi tone was provided as a further reward signal.

#### Further Feedback Considerations

Another important question is how fine-grained the feedback signal should represent changes in brain activity. This, for example, regards the resolution of the color saturation or tone frequency when computing the transfer from the EEG feature to feedback signal values. Should a feedback signal represent the neural feature proportional, or rather binary? Colgan (1977) investigated the effects proportional and binary feedback within three conditions of heart-rate-based biofeedback: only proportional feedback, only binary feedback, and a combination of the two. The authors found that proportional feedback was clearly most effective, and that the addition of a binary signal did not lead to a further increase in self-regulation. Strehl (2014) came to similar conclusions based on the neurofeedback study reported by Travis et al. (1974).

In terms of operant conditioning, an periodic reinforcement schedule would mean that the feedback signal is not continuously presented, but that a feedback on the performance is only given either after a certain number of times the targeted brain state has been reached, or in a temporally scheduled way, e.g., with a feedback signal being presented every 10 s based on the average performance within the preceding time interval. Basic science suggests that a switch from continuous to periodic reinforcement may foster training outcome (Spada et al., 2004; Thompson and Iwata, 2005), but this notion has not yet been stringently tested in context of neurofeedback learning. Related to this discussion are general delays between the recording of brain activity and the presentation of a feedback signal, which may be due to large temporal windows used for feature extraction, or time-consuming calculations during real-time processing of the EEG. The width of temporal windows should be adapted relative to the temporal characteristics of the feature of interest; low frequency as opposed to higher frequency features necessitate the extraction of larger windows, such that a given neural feature is differentially well captured with windows of different widths (e.g., Darvishi et al., 2013). Regarding the timing of feedback presentation, it has been shown temporally intermitted scheduling can be beneficiary, but also that generally delayed reinforcer may reduce the learning outcome (e.g., Skinner, 1958). When reviewing reinforcement plans, Sherlin et al. (2011) recommended the latency between neural target state and reinforcing feedback not to exceed 250–350 ms.

Related to continuous vs. discrete feedback is the post reinforcement synchronization (PRS), initially observed in an animal study by Clemente et al. (1964), and refers to alpha-like EEG synchronization in the parieto-occipital cortex after reinforcement. PRS seems to depend on the operant response (Poschel and Ho, 1972). However the PRS is also observed in humans (Hallschmid et al., 2002). Because the PRS seems positively related to learning outcome as shown in an animal study (Marczynski et al., 1981), a discontinuous or discrete feedback signal may be recommended to allow the PRS to emerge when the criteria for reinforcement are met (Sherlin et al., 2011; Strehl, 2014). An upper-alpha upregulation protocol, for example, may rely on the update of the feedback signal each time the learners’ alpha exceeds a specific threshold in the intended direction. These occasions may then be followed by a short break. In fact, Sherlin et al. (2011) suggested that discrete feedback signals as implemented by Sterman et al. (1974) may be more appropriate than the continuous forms that are used in the modern literature. Sterman et al. (1974) combined a visual (green color) and an auditory signal when the brain activity met the target state for a certain time of amount. In the case of closed-loop BCI neurofeedback paradigms, such discrete feedbacks are also regularly implemented (e.g., Ramos-Murguialday et al., 2013; Pichiorri et al., 2015). In a study of Pichiorri et al. (2015), patients with motor deficits underwent SMR training and were instructed to perform motor imagery, either imagining a grasping hand movement or a finger extension to move a virtual hand. When the brain activity met the specific criterion, Pichiorri et al. (2015) provided discrete reward in form of visually enriched feedback consistent with the imagery content: the virtual hand moved accordingly. Their results furthermore showed that functional and neurophysiological improvements correlated with the connectivity changes of oscillatory patterns.

#### Complexity of the Feedback Signal

Another issue related to the design of the feedback signal relates to the complexity of the presented stimuli. On the one hand, rather simple signals such as tones or colored geometrical shapes have been used and seem to be preferred in research settings, whereas more complex stimulus configurations such as thermometer readouts, flying rockets or videos can often be found in commercial software packages targeting clinical applications.

It is worth considering that complex stimulus configurations might induce effects on the learner that are hard to predict. For instance, using the replay of videos when brain activity is modulated according to instructions and stopping them otherwise may well have additional effects on brain processing. In fact, the putative benefit of complex or “real-world” feedback signals has not yet been studied. The amount of additional processing effects on the brain and its dissociation from neurofeedback effects, the amount of helpful reward processes for the learner, as well as the clearness and ease to understand its usage are largely unknown factors. With respect to external devices, Collura (2013) discusses possible disadvantages such as the difficulty to configure and operate such devices at a suitable timing necessary for the learning process.

Nonetheless, at least multi-stimulus feedback procedures might be well suitable when several concurrently relevant sub-goals are utilized. For example, the radius of a sphere could be used to represent the modulation towards increased activity in one, and the color of a square the decrease of activity in another frequency band.

#### Neurofeedback Software

Altogether, the previous choices determine which neurofeedback software is suited best. When a standard neurofeedback protocol is chosen to implement a therapeutic intervention in a patient population, commercial products can be a good choice. However, a disadvantage of such commercial products might be a limited flexibility with respect to the implementation of parameters such as those discussed earlier. Huster et al. (2014) provide an overview of open-source software packages based on programming features and their general purpose. Notable packages are BCI2000^1^, Open ViBE^2^, and BCILAB^3^. A software package specifically developed for BSDS, is the Constance System for Online EEG (ConSole)^4^ (Hartmann et al., 2011). Marzbani et al. (2016) introduce a neurofeedback software based on virtual reality named GRAZ-BC. A rough overview of commercial and open-source neurofeedback software packages can be found on Wikipedia^5^.

It might also be worth hinting at recent hardware developments, such as that of small, portable, and wireless products that may be of special interest for daily-life applications (De Vos et al., 2014). Furthermore, such mobile training procedures may positively impact training generalizability: if control over brain activity is learned only in a specific learning location, this ability is probably associated with the specific training environment, and is not easily retrieved or replicated in other locations (e.g., Smith et al., 1978; Smith and Rothkopf, 1984). Wireless EEG systems may be well suited for neurofeedback trainings administered outside of laboratory settings. Particularly from the view of application, small, portable and wireless products additionally increase the clinical relevance.

## Assessing the Learning Outcome and Transfer Effects

### Calculation of Learning Indices

#### Measures Assessing Changes of Brain Activity during Neurofeedback

Dempster and Vernon (2009) suggested three major measures that can be used to detect three types of brain activity changes due to neurofeedback. The first measure simply specifies absolute values of amplitude/power. The second measure specifies the percent of time spent with positive feedback, which equals the time spent in the intended brain state. Thus, this is the time spent above or beneath a specific threshold when self-regulating one’s brain activity. The third measure combines the previous two indices by calculating:

the percent time spent in the desired brain state * mean level of the amplitude during neurofeedback

Each of these measures seem to reflect different aspects of brain activity, and it might be worthy reporting all measures. To start with, over the course of training it is possible to observe changes in both amplitude and percent in time, or in one of these measures only (Dempster and Vernon, 2009). To illustrate this, the learner could exhibit only brief and temporally unstable increases over time, which nonetheless could be reflected in average amplitude changes (Hardt and Kamiya, 1976). However, the opposite could also be the case; the learner could show slight differences within training that are temporally stable. With respect to the comparison of different frequencies, it might be worth calculating these measures in both the absolute amplitude values as well as changes in percent, since amplitude scales with frequency. These measures can be used to compute individual as well as group-based learning profiles.

#### Choosing a Reference for Changes Brain Activity

Based on these measures, one can assess feature changes in four different ways: (i) within sessions, for instance by comparing the beginning of each session with the end of a training session (sessions can be arbitrarily divided into blocks or segments for statistical analyses); (ii) possible changes in these measures within a session observed as a difference relative to a baseline measurement, where participants neither try to control their brain activity nor receive any feedback; as (iii) changes observed from session-to-session; and accordingly also; as (iv) changes across sessions relative to a baseline measure.

### Training Specificity: Calculation of the Whole Frequency Spectrum

Finally, training specificity can be determined by repeating steps in Section “Measures Assessing Changes of Brain Activity during Neurofeedback” for different frequencies, assessing whether the target feature or frequency of interest has been predominantly modulated; the calculation of the whole frequency spectrum often is very informative step.

### Transfer of Training

Last but not least, the transfer of neurofeedback training assessed within pre-post measurement designs is of crucial importance (Frison and Pocock, 1992; Senn, 2007; Knapp and Schafer, 2009). Possible variables concern behavioral changes in cognitive tasks, or symptom severity in patients. However, neuroplastic changes can be induced by training, practice and learning (Kolb and Whishaw, 1998) and the contribution of Hebbian as well as homeostatic plasticity has been discussed for neurofeedback too (see Legenstein et al., 2008, 2010; Ros et al., 2014). Ghaziri et al. (2013) investigated microstructural changes in white and gray matter after 40 sessions of neurofeedback over the course of 13.5 weeks. By taking a dynamical system approach it has been suggested that the brain is operating in so called critical points, reflecting a homeostatic state enabling maximal flexibility and ability to adjust responses to various demands (Linkenkaer-Hansen et al., 2001; Chialvo, 2010). Neurofeedback could trigger the tuning of the brain’s intrinsic mechanisms of homeostasis to self-organize towards an optimal state. In a pre-post resting state design in patients with post-traumatic stress disorder, Ros et al. (2016) analyzed long-range temporal correlations of oscillations and demonstrated the reversal of abnormally random dynamics after an alpha neurofeedback training. Interestingly, this measure was correlated with improvements in symptom severity. Apart from the investigation of neuroplastic effects, the long-term stability of training-induced effects can also been investigated. Gani et al. (2008) reported reduced behavioral symptoms, improvements in cognition, and preserved EEG-regulation skills in children no longer meeting ADHD criteria.

## Illustration of a Protocol-Set Up

**Figure 5:**
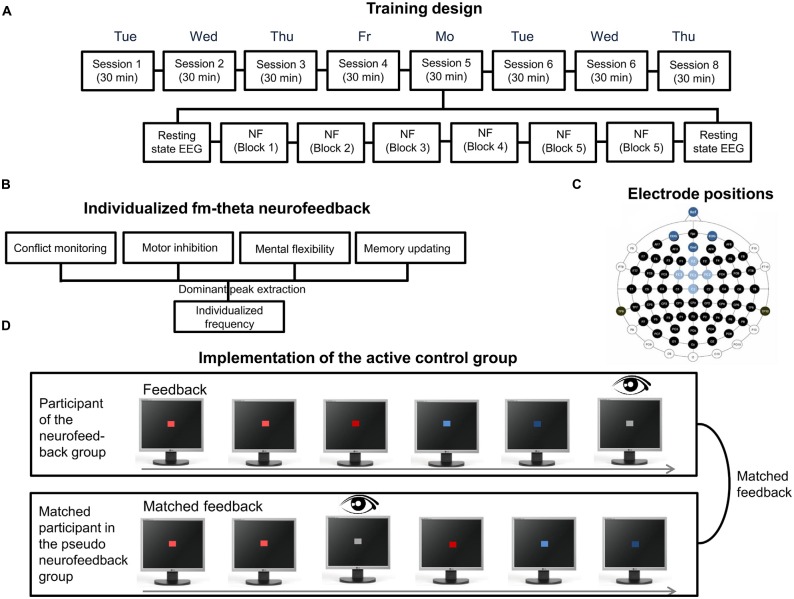
**Features of the individualized and adaptive fm-theta neurofeedback training I. (A)** Eight session training design. Each session consisted of six 5 min training blocks, which were preceded and completed by each a 5 min resting state electroencephalography (EEG) measurement. **(B)** Individualized frequency procedure. The estimation of the dominant fm-theta frequency is based on the extraction of the dominant peaks of four executive tasks. **(C)** Electrode positions. Electrode positions in light blue represent the electrodes used for neurofeedback. Dark blue represents the ground and reference electrode as well as the electrodes for EOG. **(D)** Implementation of the active control group. Each participant of the pseudo-neurofeedback group was matched to one participant of the experimental group and received his/her feedback as playback. Thereby participants of the active control group received the same visual stimulation as participants of the experimental group. To increase high credibility, participants of the active control group additionally received real eyeblink feedback.

As an example for the implementation of the steps of the decision diagram, the fm-theta neurofeedback protocol based on Enriquez-Geppert et al. (2014a,b) will serve as an example. Regarding the fm-theta protocol, ethical approval was obtained from the ethics committee of the University of Oldenburg, Germany. The aim of the fm-theta protocol was to investigate the trainability of fm-theta, and to assess the training’s effects on executive functions. Therefore, a protocol was set up including an active control group, the so-called pseudo neurofeedback group. Participants of the pseudo neurofeedback group were pseudo-randomly matched to the experimental group and received a playback of a matched participant’s feedback in the equivalent training block and training session to obtain similar sensory stimulation. Additionally, they received their own eye-blink feedback in order to increase the credibility of the pseudo-feedback manipulation (Section “Training Design”; see Figure 5D). The protocol consisted of an eight session training, with each session having a duration of 30 min (plus two resting state measures of 5 min each before and after the training; Section “Number of Training Sessions”). Each session was split up into six 5-min training blocks with self-paced breaks in between (Section “Single Session Considerations”). The training as a whole was performed over the course of 2 weeks (Section “Distribution of Neurofeedback Sessions Over the Whole Training”), whereby training sessions were completed on consecutive working days (see Figure 5A). Participants received a collection of possible strategies on how to enhance one’s own fm-theta. They were instructed to test these as well as strategies by themselves in order to select the best working mental operation to enhance their brain activity (Section “Provision of Strategies”). As the event-related fm-theta at fronto-medial electrode sites was suggested as “working language” of executive functions (Cavanagh and Frank, 2014), and was shown to be enhanced during successful processing of demanding cognitive tasks (Sederberg et al., 2003), theta frequencies at five fronto-medial electrode positions was selected for neurofeedback (Section “Feature-Extraction”; see Figure 5C). The individual dominant fm-theta peak was estimated before neurofeedback based on four tasks probing executive functions (see Figure 5B). The individual peak frequency was then used during training sessions for personalized feedback (Section “Individualization of Feature Extraction”).

Amplitude changes of theta activity during training were compared to the start-baseline measure as calculated by a Fast Fourier Transform. During training, a sliding analysis window of 2 s (Section “Defining a Threshold”) was updated every 200 ms. A simple stimulus constellation (Section “Complexity of the Feedback Signal”) was utilized for visual feedback signals (Section “Feedback Modality”). Specifically, the color saturation of a square was adapted in accordance with the ongoing theta activity (Section “Further Feedback Considerations”). Red corresponded to theta activity that was enhanced relative to the start-baseline, and blue to reduced activity. NeuroFeedback Suite 1.0 (Huster et al., 2014) was selected as software. It has the advantage of representing a ready-to-use neurofeedback software with the unique feature of multiple-subject data management (Section “Neurofeedback Software”). Thereby, single or double-blinded studies can easily be performed. It furthermore includes a template for individualized eye-artifact removal. Both absolute and relative amplitude changes of theta over the course of the training were computed (Section “Measures Assessing Changes of Brain Activity during Neurofeedback”). In addition, to further evaluate the training specificity of fm-theta neurofeedback, the neighboring frequencies, alpha and beta bands, were also assessed statistically (Section “Transfer of Training”). The whole frequency spectrum was inspected and compared before and after neurofeedback.

**Figure 6:**
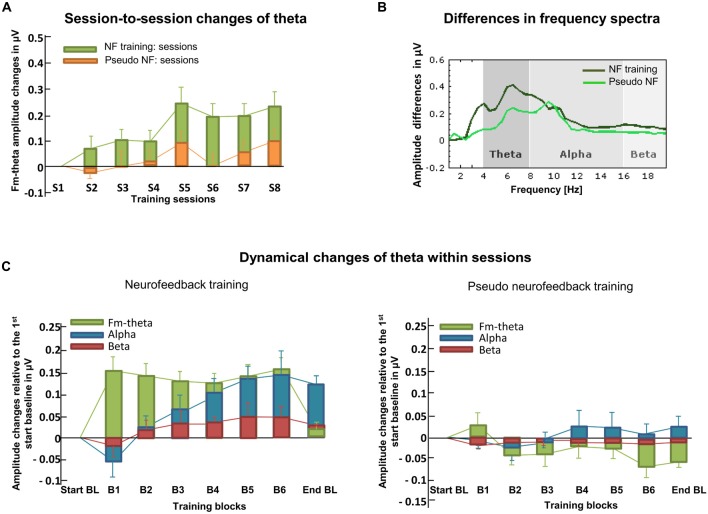
**Learning indices. (A)** Here, session-to-session changes during neurofeedback are illustrated as calculated for theta frequencies. Stronger increases in the actual neurofeedback intervention are visible compared to the pseudo neurofeedback intervention. **(B)** The frequency spectra depict the amplitude changes of theta, alpha and beta from the first to the last training session for both, the neurofeedback- and the pseudo neurofeedback training group. **(C)** The dynamical changes within sessions and across all training days are illustrated for theta, alpha and beta recorded in each training block for both the neurofeedback- and the pseudo neurofeedback training group. Based on real feedback, only the neurofeedback training group shows enhanced theta that is not visible in the active control group (adapted from Enriquez-Geppert et al., 2014b).

As can be seen in Figure 6A, the session-to-session changes of theta activity were analyzed (one of three possible learning indices; Section “Choosing a Reference for Changes Brain Activity”). This graphic visualizes that proper neurofeedback training led to increased fm-theta activity when compared to pseudo neurofeedback training. The dynamical changes of theta within sessions (Figure 6C; Section “Choosing a Reference for Changes Brain Activity”) were computed as the average of each training block across the eight training sessions. This graphic demonstrates that proper neurofeedback training (left side), as compared to the pseudo neurofeedback (right side), led to an enhancement of fm-theta during training blocks. This shows that a real-time feedback serves as learning signal that can be used to self-regulate one’s own theta activity. Finally, full frequency spectra display the amplitude changes of theta, alpha, and beta frequencies from the first training session to the last, which is important regarding the evaluation of the specificity of a training (Section “Training Specificity: Calculation of The Whole Frequency Spectrum”; see Figure 6B). It can be seen that theta enhancement represents a frequency-specific training effect. Furthermore, transfer of the training to tasks assessing four executive functions (conflict monitoring and motor inhibition, memory updating and mental flexibility were found (Cavanagh and Frank, 2014). Fm-theta neurofeedback did not affect reactive control mechanisms (Stroop and Stop Signal tasks), but facilitated proactive control (as indexed by the three-back task and the task-switching task (Section “Training Specificity: Calculation of the Whole Frequency Spectrum”; see original report in Enriquez-Geppert et al., 2014a).

In the following paragraphs, the prerequisites for the interpretation and assessment of neurofeedback, and training criteria as well as potential guidelines for the evaluation of training efficacy in the clinical domain are assessed. Last but not least, a framework for further development of neurofeedback protocols is discussed.

## Discussion

**Figure 7:**
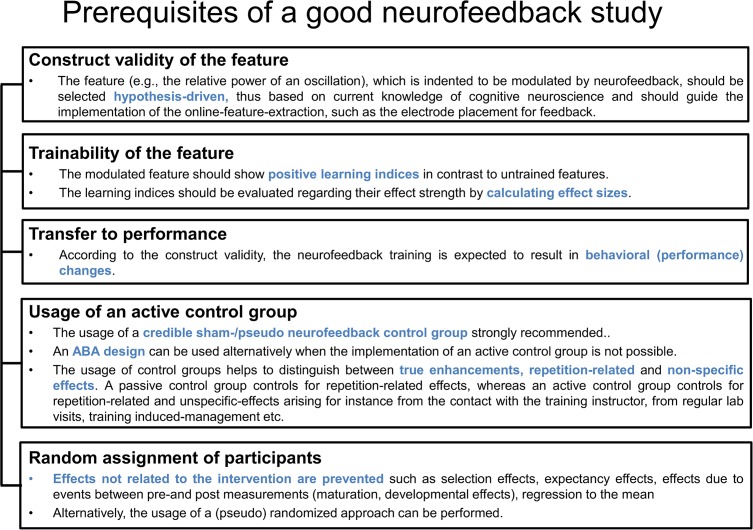
**Prerequisites of the neurofeedback design.** This figure lists four criteria for the validation of neurofeedback studies and refers to the interpretability of trained features, the trainability of the feature, the usage of an active control group and the random assignment of participants.

Regarding the assessment of the outcome of neurofeedback training protocols, a debate trying to define best-practice guidelines has just started (Gruzelier, 2014b; Strehl, 2014). Of crucial importance for the interpretation and assessment of neurofeedback results are prerequisites concerning the design. This includes the usage of an active control group implemented by a credible sham-/pseudo neurofeedback group, in order to dissociate between true as compared to repetition-related or non-specific effects. Of similar importance is the random assignment of participants to experimental and control groups to prevent effects not related to the specific neurofeedback protocol such as selection or expectancy effects (see Figure 7 for an overview). Meeting these criteria will provide a good basis for a well-designed neurofeedback study.

### Prerequisites of a Good Neurofeedback Study

Neurofeedback-specific principles are presented in the following text that should be considered prerequisites to allow conclusions regarding the training efficacy. These principles refer to the construct validity of the feature selection, the trainability of the feature itself, as well as behavioral and neurocognitive transfer effects (see Figure 7). **Construct validity (1)** of the feature refers to the empirically confirmed relation between the feature and a specific cognitive function or symptom that is intended to be changed by neurofeedback. The feature thus represents a crucial underlying neural mechanism of a cognitive process of interest. The second aspect that is specific to the evaluation of neurofeedback refers to the **trainability (2)** of the feature as reflected in appropriate learning indices. The assessment of the trainability of a feature should further be accompanied by the calculation of effect sizes to make more precise quantitative statements. The specificity of the training outcome is highest with modulations seen predominantly (or even exclusively) with the trained feature, without affecting untrained brain processes. This aspect can also be regarded as part of construct validity. **Transfer effects according to the construct validity (3)** refer to effects on behavior as expected based on the relation between the feature and cognitive functions.

### Assessment Criteria for the Evaluation of Clinical Interventions

**Figure 8:**
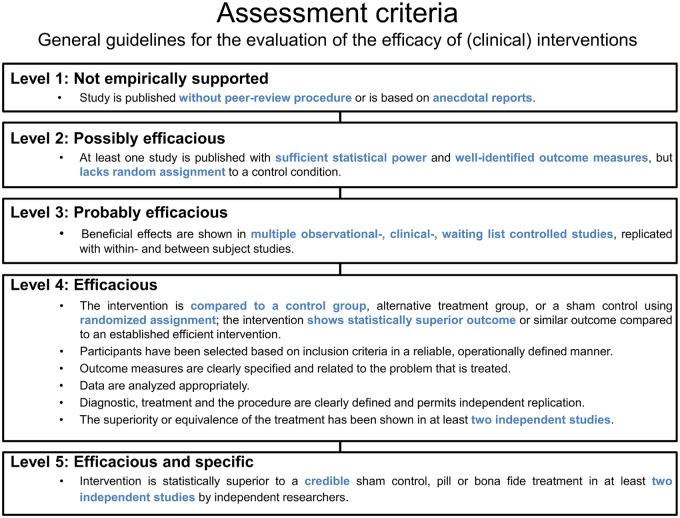
**Assessment criteria.** Based on general guidelines, five levels and their criteria are listed for the evaluation of the efficacy of (clinical) interventions.

The evaluation of neurofeedback protocols is particularly decisive with respect to their clinical efficacy. Based on an existing categorization of the American Psychological Association (APA), guidelines (LaVaque et al., 2002) have been developed by collaborative work of two neurofeedback societies (the Association for Applied Psychophysiology and Biofeedback (AAPB) and the Society for Neuronal Regulation (SNR)). These guidelines specify rules for the assessment of treatment evidence as summarized in Figure 8. Five different levels are differentiated that classify the efficacy of an intervention. The lowest level (Level 1) refers to rather anecdotal or narrative reports about a seemingly effective treatment. Studies that are published without peer-review and thereby miss the opportunity to subject the work to other experts for maintaining quality standards are also regarded as Level 1 studies. Altogether, these studies are categorized as “not empirically supported”. In contrast, an intervention can be classified as “efficacious and specific” (Level 5) whenever a treatment utilizes a credible sham therapy, pill, or alternative* bona fide* treatment in at least two independent research settings, and furthermore meets the demands of the lower levels (e.g., sufficient statistical power, a well-defined outcome measure, an appropriate control group, replicability; see Figure 8).

### Outlook

EEG-based neurofeedback represents a non-invasive, economical, and potentially mobile technique for the modulation of brain activity. The previously discussed elements that constitute a feedback system also provide a framework for the discussion of further development. For instance, during online data-preprocessing, most studies deal with eye artifacts by online rejection, thereby disrupting the quasi-continuous stream of the learning signal that otherwise might easily be contaminated with artifacts. Through the rejection of these time frames the available learning time is also reduced during neurofeedback sessions. This might be additionally challenging with clinical populations such as patients with increased motor agitation, or whenever medication produces side effects leading to increased and uncontrolled movements. Thus, an adaptation of correction procedures for real-time application during neurofeedback would be advantageous (for instance by means of independent component analysis). Similarly, online feature-extraction could significantly be improved by using advanced signal processing routines. Scalp EEG recordings necessarily reflect a mixture of activities from multiple brain sources. Thus, the application of source-based signal processing for neurofeedback may significantly increase the specificity and efficacy of a neurofeedback training protocol. Correspondingly, White et al. (2014) recently presented a study that used such advanced methods for EEG neurofeedback in order to self-regulate theta oscillations originating from medial-temporal and parietal regions.

In summary, a well-designed neurofeedback system relies on the characteristics of five processing elements in order to optimize the self-regulation of brain activity and enable transfer to cognition and behavior. Based on the neurofeedback design and criteria concerning the evaluation of clinical efficacy, concrete conclusions regarding training results are facilitated. Despite a number of improvements that still need to be applied more widely to common protocols, EEG neurofeedback represents a feasible and promising tool for therapeutic interventions, cognitive enhancement, as well as a method for basic research.

## Author Contributions

SE-G, RJH and CSH are responsible for the conception of the work. SE-G and RJH drafted the article and created the figures. SE-G wrote the article. RJH and CSH revised the article. SE-G, RJH and CSH approved the version to be published.

## Conflict of Interest Statement

The authors declare that the research was conducted in the absence of any commercial or financial relationships that could be construed as a potential conflict of interest. The reviewer SLGA and handling Editor declared their shared affiliation, and the handling Editor states that the process nevertheless met the standards of a fair and objective review.

## Footnotes

^1^http://www.bci2000.org

^2^http://openvibe.inria.fr

^3^http://sccn.ucsd.edu/wiki/BCILAB

^4^http://console-kn.sf.net

^5^https://en.wikipedia.org/wiki/Comparison_of_neurofeedback_software
